# The miR-26b-5p/KPNA2 Axis Is an Important Regulator of Burkitt Lymphoma Cell Growth

**DOI:** 10.3390/cancers12061464

**Published:** 2020-06-04

**Authors:** Fubiao Niu, Marta Kazimierska, Ilja M. Nolte, Miente Martijn Terpstra, Debora de Jong, Jasper Koerts, Tineke van der Sluis, Bea Rutgers, Ryan M. O’Connell, Klaas Kok, Anke van den Berg, Agnieszka Dzikiewicz-Krawczyk, Joost Kluiver

**Affiliations:** 1 Department of Pathology and Medical Biology, University of Groningen, University Medical Center Groningen, 9700RB Groningen, The Netherlands; f.niu@umcg.nl (F.N.); d.de.jong03@umcg.nl (D.d.J.); j.a.koerts@umcg.nl (J.K.); t.van.der.sluis@umcg.nl (T.v.d.S.); b.rutgers@umcg.nl (B.R.); a.van.den.berg01@umcg.nl (A.v.d.B.); 2 Institute of Human Genetics, Polish Academy of Sciences, 60-479 Poznan, Poland; marta.kazimierska@igcz.poznan.pl; 3 Department of Epidemiology, University of Groningen, University Medical Center Groningen, 9700RB Groningen, The Netherlands; i.m.nolte@umcg.nl; 4 Department of Genetics, University of Groningen, University Medical Center Groningen, 9700RB Groningen, The Netherlands; m.m.terpstra.cluster@gmail.com (M.M.T.); k.kok@umcg.nl (K.K.); 5 Division of Microbiology and Immunology, Huntsman Cancer Institute, Department of Pathology at the University of Utah, Salt Lake City, UT 84112, USA.; ryan.oconnell@path.utah.edu

**Keywords:** Burkitt lymphoma, miR-26b-5p, microRNA, KPNA2

## Abstract

The expression of several microRNAs (miRNAs) is known to be changed in Burkitt lymphoma (BL), compared to its normal counterparts. Although for some miRNAs, a role in BL was demonstrated, for most of them, their function is unclear. In this study, we aimed to identify miRNAs that control BL cell growth. Two BL cell lines were infected with lentiviral pools containing either 58 miRNA inhibitors or 44 miRNA overexpression constructs. Eighteen constructs showed significant changes in abundance over time, indicating that they affected BL growth. The screening results were validated by individual green fluorescent protein (GFP) growth competition assays for fifteen of the eighteen constructs. For functional follow-up studies, we focused on miR-26b-5p, whose overexpression inhibited BL cell growth. Argonaute 2 RNA immunoprecipitation (Ago2-IP) in two BL cell lines revealed 47 potential target genes of miR-26b-5p. Overlapping the list of putative targets with genes showing a growth repression phenotype in a genome-wide CRISPR/Cas9 knockout screen, revealed eight genes. The top-5 candidates included EZH2, COPS2, KPNA2, MRPL15, and NOL12. EZH2 is a known target of miR-26b-5p, with oncogenic properties in BL. The relevance of the latter four targets was confirmed using sgRNAs targeting these genes in individual GFP growth competition assays. Luciferase reporter assay confirmed binding of miR-26b-5p to the predicted target site for KPNA2, but not to the other genes. In summary, we identified 18 miRNAs that affected BL cell growth in a loss- or gain-of-function screening. A tumor suppressor role was confirmed for miR-26b-5p, and this effect could at least in part be attributed to KPNA2, a known regulator of OCT4, c-jun, and MYC.

## 1. Introduction

Burkitt lymphoma (BL) is a highly aggressive B-cell lymphoma that was first reported in 1958 in Uganda. Three main clinical variants of the Burkitt lymphoma are recognized, the endemic variant that is associated with the Epstein–Barr virus (EBV), the sporadic type, and the immunodeficiency-associated variant that is usually associated with HIV or occurs in post-transplant patients [1]. This malignancy mainly affects children and young adults and originates from germinal center (GC-) B cells [2,3,4,5]. The hallmark of BL is a chromosomal translocation involving MYC and the immunoglobulin heavy or light chain gene loci, which results in increased expression of MYC [6].

MicroRNAs (miRNAs) are a group of small, non-coding RNAs that regulate gene expression, mainly at the post-transcriptional level, through inhibition of translation or RNA degradation [7]. MiRNAs regulate almost all known cellular processes, including B-cell maturation and development, cell proliferation, cell cycle, and apoptosis [8]. MiRNA expression profiling showed distinct expression patterns in different subtypes of B-cell lymphomas [9]. Multiple miRNAs were deregulated in BL, as compared to the GC-B cells and other types of B-cell lymphomas [9,10,11,12,13]. We previously reported 65 miRNAs that were differentially expressed between sporadic BL and normal GC-B cells, and identified several of these miRNAs as MYC-regulated [13]. Others have shown 49 miRNAs that were differentially expressed in endemic BL, as compared to normal GC-B cells [12]. Profiling of the three clinical BL variants in comparison to diffuse large B cell lymphoma (DLBCL), revealed highly similar miRNA profiles between the three BL variants with 38 miRNAs that differentiated BL from DLBCL [11]. Another, more extensive study revealed a total of 35 deregulated miRNAs in BL, compared to seven other types of non-Hodgkin lymphomas [9]. Comparison between BL, follicular lymphoma (FL), and DLBCL, revealed 22 miRNAs that could separate BL from the other two lymphoma subtypes [10]. Functional follow-up studies revealed that some of the differentially expressed miRNAs had oncogenic (miR-17~92 cluster) or tumor suppressor properties (miR-150-5p, miR-28-5p, and miR-26-5p) in BL [14,15,16,17,18]. However, for most of the deregulated miRNAs, the target genes relevant for BL pathogenesis and the mode of action remain unknown.

In this study, we identified 18 miRNAs involved in controlling BL cell growth in a high-throughput loss- and gain-of-function screen. We further studied the relevance of miR-26b-5p and its target genes. Overexpression of miR-26b-5p strongly decreased the growth of BL. Immunoprecipitation of the Ago2-containing RISC (Ago2-IP) and overlap with results of a genome-wide CRISPR/Cas9 screening, identified eight putative miR-26b-5p target genes whose knockdown resulted in impaired growth. Four candidates were confirmed as essential genes for BL cell growth and the predicted miR-26b-5p binding site was confirmed for KPNA2.

## 2. Results

### 2.1. Validation of the Efficiency of the miRNA Inhibition and Overexpression Pools

To verify the expression of the functional antisense miRNA strands of the miRZip inhibition constructs, we performed small RNA sequencing on the ST486 cells infected with the miRZip pool (including 55/60 miRZip constructs). This revealed that for 52 of the 55 constructs, the antisense miRZip-3p (functional) strand was detected at >150 reads per million (RPM) (range 165 to 33,411). For 48 of the 55 constructs, the miRZip-3p strand was more abundant than the miRZip-5p strand (Figure S1A). Thus, the vast majority of the miRNA inhibition constructs showed a predominant expression of the antisense strand with relatively high levels.

The effectiveness of the constructs in the pCDH pool was validated by small RNA sequencing of pCDH pool-infected HEK-293T cells (including 39 pCDH constructs). HEK-293T cells were used to obtain a high infection percentage, which was not possible for the BL cell lines. Four of the 78 potential mature miRNAs derived from the 39 miRNA overexpression constructs were not reported in the miRBase (http://www.mirbase.org) and were also not detected in the small RNA sequencing data. For 36 of the 39 constructs, we detected the most abundant strand at an RPM of >150 reads (range 190 to 40,834). For 19 constructs, miRNAs from the most abundantly expressed strand showed >2-fold increased levels in the pCDH pool-infected HEK-293T cells, as compared to the empty vector infected HEK-293T cells; four miRNAs showed a moderate increase between 1.5- to 2-fold; and seven showed a slight increase ranging from 1.0- to 1.5-fold. The remaining nine constructs showed no change or a slight decrease (Figure S1B). In total, 30 out of 39 (77%) miRNA overexpression constructs showed increased expression of the corresponding miRNAs in the HEK-293T cells.

Thus, most constructs present in the miRNA inhibition and overexpression pools showed the expected expression pattern. As these data were generated from the cells infected with the lentiviral pools of 55 or 39 constructs, with each construct being present in a minority of the cells, we anticipated that the observed efficiencies were in fact an underestimation of the actual performance of the constructs.

### 2.2. Identification of miRNAs Affecting the Growth of BL Cells

**Figure 1:**
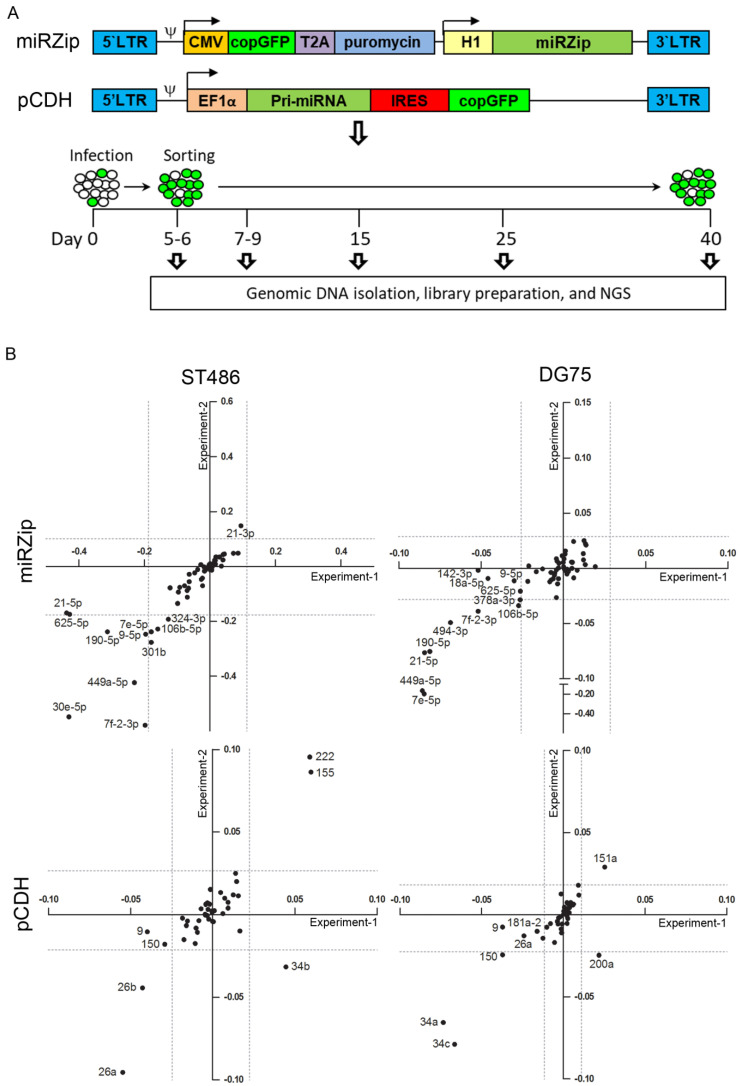
Overview of the high-throughput screening and identification of miRNAs affecting the BL cell growth in the miRZip and pCDH pools. (**A**) Overview of the miRNA inhibition (miRZIP) and overexpression (pCDH) vectors and the workflow of the high-throughput screen. (**B**) miRZip and pCDH constructs with significant changes in abundance over time in BL cell lines ST486 and DG75. Construct abundance was represented by the slope of the trend line calculated on the basis of the relative fold changes, for all time points relative to day 5 (miRZip) or 6 (pCDH). The X-axis and Y-axis represent the slopes of the trend lines of duplicate infections (negative values = decreased abundance, positive values = increased abundance). Dashed lines represent the cut-off for significant changes in abundance, calculated by an adapted interquartile (IQR) test.

To identify miRNAs relevant for BL cell growth, we conducted a screen with miRNA overexpression and inhibition libraries in the ST486 and DG75 cells (Figure 1A). The infection efficiencies achieved for the virus pools were ~10–15% at the starting point of the experiments (day 5 or 6), which corresponded to 0.75 to 1.13 million infected (GFP+) cells. The GFP+ cell percentages in the sorted fractions were at least 45% for the pCDH infected cells and at least 75% for the miRZip infected cells (Figure S2A,C). Effective amplification of the barcode inserts was tested on agarose gel and the PCR products were pooled for next generation sequencing (NGS) (Figure S2B,D). Total read counts obtained by NGS ranged from about 30,000 to 150,000 for miRZip samples and from about 20,000 to 100,000 for the pCDH samples (Figure S2E, Tables S1 and S2). Average read numbers per PCR replicate of each construct were calculated and eleven constructs (5 of 60 miRZip constructs and 6 of 46 pCDH constructs) with average reads <50 at the first time point, were excluded from the downstream analysis (Figure S2F,G).

Abundance of the negative controls in both the miRZip and pCDH pools remained stable over time. In the miRZip pool, we identified ten miRNA inhibitors with consistently decreased abundance in duplicate infections in ST486 or DG75 (Figure 1B). MiRZip-let-7f-2-3p, miRZip-190-5p, and miRZip-449a-5p showed a consistent decrease in abundance in both cell lines, while miRZip-9-5p, miRZip-106b-5p, miRZip-21-5p, miRZip-let-7e-5p, miRZip-494-3p, miRZip-30e-5p, and miRZip-378a-3p were depleted in one cell line. None of the miRZip constructs showed a consistent increase in abundance over time.

In the pCDH pool, consistent decreases in abundance were observed for five constructs and consistent increases were observed for three constructs (Figure 1A,B). pCDH-miR-26a and pCDH-miR-26b were depleted in ST486, while pCDH-miR-34a, pCDH-miR-34c, and pCDH-miR-150 were depleted in DG75. Increased abundance was observed for pCDH-miR-155 and pCDH-miR-222 in ST486, and for pCDH-miR-151a in DG75. The effects of overexpression of miR-155, miR-150, miR-26a, and miR-26b on BL cell growth were in line with our previous findings [16,19]. In summary, we identified 13 miRNAs (10 from the miRNA inhibition and 3 from the miRNA overexpression screen) that stimulate BL cell growth and 5 that repress BL cell growth (all from the overexpression screen).

### 2.3. Selection of MiRNA Candidates for Functional Studies

**Figure 2:**
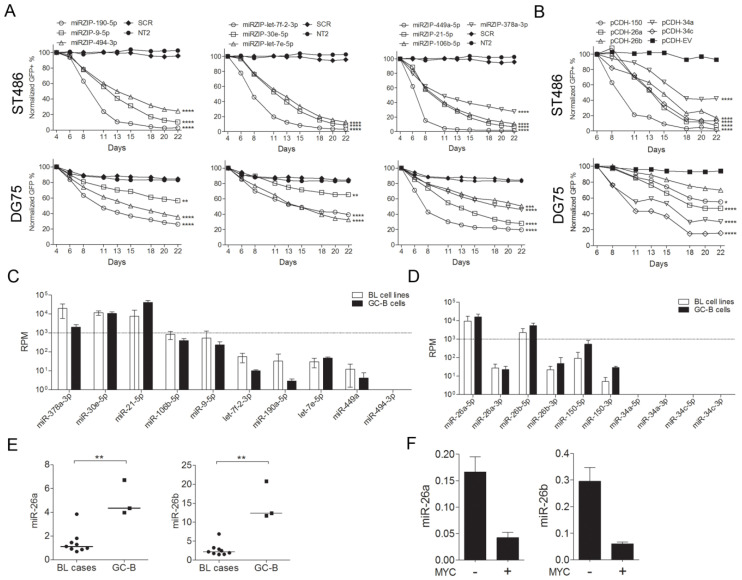
Validation of screening results and selection of miRNAs for functional experiments. Green fluorescent protein (GFP) growth competition assay of individual (**A**) miRNA inhibition and (**B**) miRNA overexpression constructs in ST486 and DG75 cells. The GFP+ cell percentage was measured using flow cytometry for 22 days, and the percentage at the first day of measurement was set to 100%. * *p* < 0.05, ** *p* < 0.01, *** *p* < 0.001, and **** *p* < 0.0001, based on mixed-model analysis. (**C**) Small RNAseq-based expression levels of the miRNA candidates from the miRZip pool and the (**D**) pCDH pool. Shown are the average read counts of 4 BL cell lines (ST486, CA46, DG75, and Ramos) and the average of 3 GC-B cell samples. (**E**) Expression of miR-26a/b-5p in the BL tissue samples and the GC-B cells, and in (**F**) the P493-6 B-cells, with and without MYC expression relative to RNU49 by RT-qPCR. ** *p* <0.01 (Mann-Whitney U-test). The lines represent the median of the miRNA levels.

To validate the results of the high-throughput screen, GFP growth competition assays were performed using the 10 miRZip and 5 pCDH constructs, which showed a decrease in abundance in the screen. A decrease in the GFP+ cell population was observed for all constructs in both BL cell lines. Thus, constructs that were significantly depleted in only one of the two BL cells lines in the screens, affected growth in both cell lines, using individual GFP growth competition assays (Figure 2A,B).

For further functional analysis, we selected miRNAs with a differential expression between the BL and the GC-B cells, based on small RNA sequencing data [20]. As a second criterion, we focused on miRNAs with a relative abundance of approximately 1,000 RPM in the BL cell lines (for the miRZip constructs) or in the GC-B cells (for the pCDH constructs). This RPM criterion was based on the results of a high-throughput assessment of miRNA activity [21] (Figure 2C,D). These two criteria resulted in four miRNA candidates, i.e., miR-378a-3p from the miRZip pool and miR-150-5p, miR-26a-5p, and miR-26b-5p from the pCDH pool. As we previously studied the function of miR-378a-3p [20] and miR-150-5p in BL [16], we decided to further focus on miR-26a-5p and miR-26b-5p, two seed members of the miR-26-5p family.

To confirm the decrease in miR-26a-5p and miR-26b-5p expression in BL cell lines compared to the GC-B cells, as observed by small RNA sequencing, we assessed the expression of these two miRNAs in primary BL tissues relative to the GC-B cells by qRT-PCR. Significantly decreased levels were observed for both miR-26a-5p and miR-26b-5p in the BL tissues (Figure 2E). We also confirmed the previously reported regulation of both miRNAs by MYC in the P493-6 B-cell [22]. Both miRNAs showed ≥3-fold reduction in expression upon MYC induction (Figure 2F). Taken together, miR-26a-5p and miR-26b-5p are MYC-repressed miRNAs with decreased expression in BL that upon overexpression repress BL cell growth.

### 2.4. Identification of Targets of the miR-26-5p Family by Ago2-RIP-Chip

To identify the target genes relevant for the observed effect of the two miR-26-5p family members on the BL cell growth, we carried out Ago2-RIP-Chip analysis in two BL cell lines. As both miR-26a-5p and miR-26b-5p share the same seed sequence and have a >90% overall sequence homology (Figure S3A), they are likely to target a highly similar set of genes. Therefore, we used the pCDH-miR-26b construct as being representative for both and used the pCDH-EV as a negative control (Figure S3B). The efficiency of the Ago2-IP procedure was confirmed by showing enrichment of the Ago2 protein and of miR-26-5p in the Ago2-IP fractions (Figure S3C,D, Figure S4).

**Figure 3:**
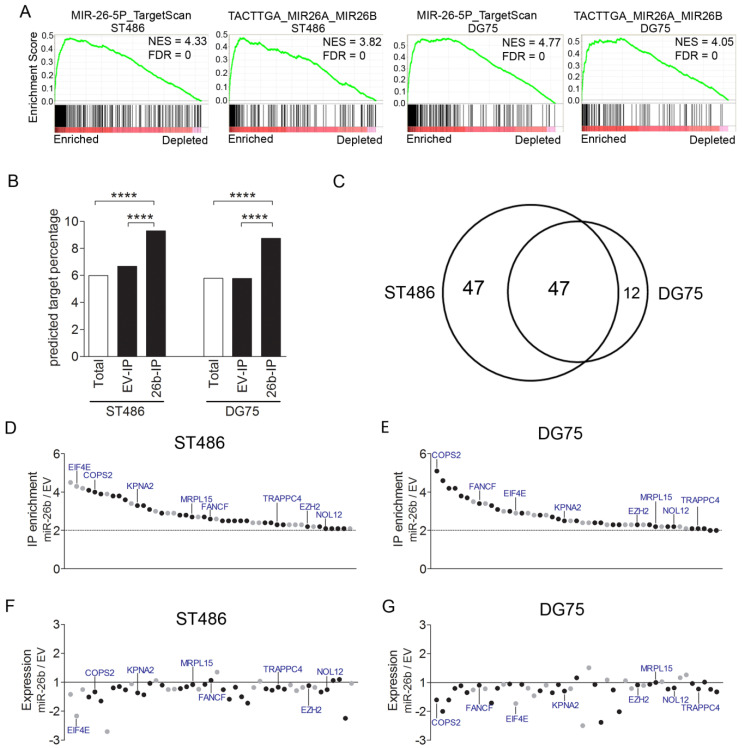
Identification of the miR-26b-5p target genes. (**A**) Enrichment plots of gene sets for the miR-26-5p targets. Pre-ranked gene set enrichment analysis was performed on average miR-26b IP/T, over empty vector (EV) IP/T fold-change values. (**B**) Comparison of the percentage of TargetScan-predicted targets of miR-26b-5p among the consistently expressed genes, and Ago2-IP enriched genes. (**C**) The overlap of the miR-26b-5p targets identified in ST486 and DG75, upon miR-26b overexpression. **** *p* < 0.0001. (**D**,**E**) IP/T ratios of the miR-26b-5p targets identified by Ago2-RIP-Chip upon miR-26b-5p overexpression, relative to EV in ST486 and DG75. The black dots indicate genes with predicted miR-26b-5p binding sites. (**F**,**G**) Fold changes in expression of the 47 genes in ST486 and DG75, upon miR-26b-5p overexpression, compared to the EV control. Same order of genes as in D and E. The 8 genes indicated in panels D to G are identified in the CRISPR/Cas9 screen shown in Figure 4A.

Analysis of the microarray data from the Ago2-IP (IP) and total (T) samples revealed 12,286 and 9,206 consistently expressed probes in ST486 and DG75, respectively. In pCDH-miR-26b and pCDH-EV-infected ST486 and DG75 cells, 1277 to 1826 probes were IP-enriched with an IP/T ratio ≥2, i.e., 13.9–15.2% of the consistently expressed probes (Table 1). Gene set enrichment analysis revealed a significant enrichment of two sets of predicted miR-26b-5p target genes in the Ago2-IP fractions of cells with miR-26b-5p overexpression (Figure 3A). These gene sets ranked as the first and second gene sets in both BL cell lines and further indicated a high efficiency of the Ago2-IP procedure. Furthermore, TargetScan-predicted miR-26b-5p targets were significantly enriched in the Ago2-IP fractions upon miR-26b overexpression, as compared to the Ago2-IP fractions of pCDH-EV in both BL cell lines (Figure 3B).

Comparing the Ago2-IP-enriched target genes of pCDH-miR-26b infected cells to those of the pCDH-EV infected cells, revealed 94 genes with a >2-fold increased enrichment in ST486 and 59 in DG75, with an overlap of 47 (Figure 3C, Table 2). The identified targets included two proven miR-26b-5p targets, i.e., *EZH2* and *KPNA2*, and one proven miR-26a-5p target, i.e., *PRKCD* [23,24,25,26,27]. The majority of the genes identified in only one of the two cell lines showed a similar increased enrichment also in the other cell line, although it did not reach our 2-fold criterion. Thirty of the 47 overlapping miR-26b-5p targets (64%) had at least one potential miR-26b-5p binding site (7mer-A1, 7mer-m8, or/and 8mer) (Figure 3D,E, and Table 2). Moreover, the vast majority of the 47 genes showed decreased expression levels upon miR-26b-5p overexpression (Figure 3F,G). In summary, using Ago2-RIP-Chip, we identified 47 putative target genes of miR-26b-5p in the BL cell lines.

### 2.5. COPS2, NOL12, MRPL15, and KPNA2 Are Potential Targets of miR-26b-5p and the Essential Genes for the BL Cells

**Figure 4:**
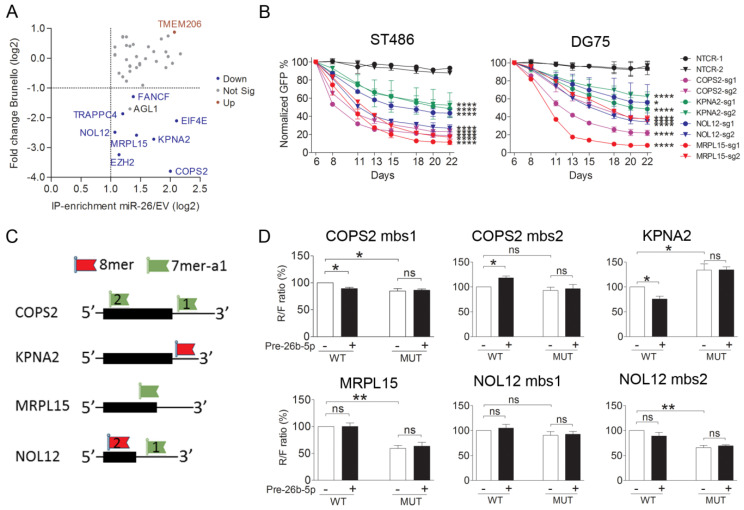
Validation of miR-26b-5p target genes. (**A**) Scatter plot displaying the results of the Brunello screen in ST486 for the identified 42 consistent miR-26b-5p target genes relative to their enrichment in the Ago2-IP fraction. The gene names indicated in the figure were identified as targets of miR-26b-5p using Ago2-IP, and showed significant changes in abundance in the Brunello screen. (**B**) Green fluorescent protein (GFP) growth competition assay with sgRNAs targeting the selected miR-26b-5p target genes in ST486 and DG75 confirm their effects on cell growth. * *p* < 0.05 and **** *p* < 0.0001, based on mixed model analysis. BL cell lines were infected with sgRNAs in duplicates, except for MRPL15-sg2, which was infected once in ST486. (**C**) Schematic representations of the location of the predicted miR-26b-5p binding sites (mbs) for the four genes selected for validation by luciferase reporter assay. The black boxes indicate the position of the open reading frames (ORF). Positions and types of miR-26b-5p binding sites are indicated, relative to the ORF. (**D**) Luciferase reporter assay results upon co-transfection of the ST486 cells with the Psi-check-2 construct containing the wildtype (WT) or mutated (MUT) miR-26b-5p binding sites from the selected genes and either miR-26b-5p mimic or a negative control mimic. Significant differences were calculated using a paired *t*-test. * *p* < 0.05, ** *p* <0.01. ns: no significance.

To aid selection of the relevant target genes for further analysis, we used data from our genome wide CRISPR/Cas9 dropout screen (in which each gene is targeted by four sgRNAs) in ST486 cells (Figure S5) [28]. Among the 47 potential targets of the miR-26b-5p common in both ST486 and DG75, 42 were included in the screen. The five genes that were not included in the screen are non-coding genes. Single guide RNAs targeting eight genes, including the two known miR-26b-5p targets, i.e., EZH2 and KPNA2, showed a significant depletion in the dropout screen (Figure 4A). Of these eight, we selected the five genes with the strongest depletion (FC < −5.0) for further validation. As EZH2 proved to be an essential gene for BL cells and a previously reported target of miR-26b-5p [29], we focused on the remaining four genes, i.e., COPS2, NOL12, MRPL15, and KPNA2.

The negative effect of the knockout of these four genes on BL cells was confirmed using CRISPR/Cas9-based GFP growth competition assays in ST486 and DG75 (Figure 4B). Compared to the negative controls, significant decreases in the number of GFP+ cells were observed for all sgRNAs targeting the four selected genes, both in ST486 and DG75. This indicated that depletion of these genes is disadvantageous for BL cells, which was in line with the phenotype induced upon miR-26b-5p overexpression.

To confirm targeting by miR-26b-5p, we performed luciferase reporter assays in ST486 for six predicted miR-26b-5p binding sites in these four genes (Figure 4C). A significant reduction in the Renilla over the Firefly (R/F) ratio upon miR-26b-5p overexpression was observed for the miR-26b-5p binding site in KPNA2 (Figure 4D) but not for the mutated site, suggesting a direct targeting of KPNA2 by miR-26b-5p. Moreover, the R/F ratio for the mutated miR-26b-5p binding site was increased, compared to the wildtype binding site in BL cells without miR-26b overexpression, indicating regulation by endogenous miR-26b-5p. A minor reduction in the R/F ratio was shown for one of the two predicted miR-26b-5p binding sites in COPS2, indicating a possible regulation by miR-26b-5p. For the remaining two genes, binding of miR-26b-5p to the predicted binding sites could not be confirmed.

## 3. Discussion

In this study, we identified 18 miRNAs that control BL cell growth, using a high throughput loss- and gain-of-function screening approach. We focused our functional follow-up experiments on miR-26a-5p and miR-26b-5p, both of which showed a negative effect on the BL cell growth upon overexpression. Knockdown of eight of the miR-26b-5p target genes identified by Ago2-IP, also inhibited the BL cell growth in a genome wide CRISPR/Cas9 dropout screen. Targeting of KPNA2 by miR-26b-5p was subsequently confirmed by the luciferase reporter assay.

In the loss-of-function screen (miRZip), ten miRNA inhibitor constructs showed a negative effect on the BL cell growth, while none of the constructs enhanced the BL cell growth. This imbalance might be explained by the selection of miRNAs included in this library. Most miRNAs were included based on previously reported oncogenic properties or enhanced expression in BL. The levels of five of the ten miRNAs, i.e., let-7f-2-3p, miR-190-5p, let-7e-5p, miR-449a-5p, and miR-494-3p were low in the BL cells, with an RPM <100 and based on a previous study, were less likely to be functionally relevant [21]. Nevertheless, we did confirm an effect on BL growth for these five miRNAs. For let-7e-5p, this might be explained by the presence of two other seed family members that showed a high expression in BL, i.e., let-7a-5p and let-7f-5p. Thus, our miRZIP constructs might not only inhibit a specific miRNA but also its closely related family members with highly similar sequences. For the four other low abundant miRNAs, the underlying explanation is less clear. However, three of them were previously indicated in B-cell lymphoma growth. A negative effect of inhibition of let-7f-2-3p and miR-449a-5p was observed in a high throughput screen in Hodgkin lymphoma [30]. MiR-494-3p was proposed to influence proliferation by inhibiting MYC expression in B-cell lines [17]. The other five candidates had expression levels that were considered to be more physiologically relevant. We recently studied miR-378a-3p and showed its involvement in BL growth [20]. An oncogenic role for miR-21-5p has been shown in many cancers and we reported its role on proliferation of the Hodgkin lymphoma [30]. In contrast to the potential oncogenic role of miR-30e-5p in our study, other seed family members have been reported to function as tumor suppressors in colorectal cancer, breast cancer, and non-small cell lung cancer [31,32,33]. An oncogenic role was shown for miR-106b-5p in non-small cell lung cancer, renal cell carcinoma, and glioma tumor [27,34,35]. Interestingly, miR-9-5p was shown to have both tumor suppressor and oncogenic properties in different cancers [36,37]. In HL, it was shown that miR-9-5p impaired tumor outgrowth in a xenograft model of HL [38].

In the miRNA overexpression library screen (pCDH), three constructs showed a positive effect on the BL cell growth and five constructs showed a negative effect. For one of the three overexpression constructs (pCDH-miR-155) with a positive effect on growth, we previously demonstrated that targeting of NIAM1 might at least in part explain the growth promoting effect of miR-155 in BL [19]. For miR-151a-3p, a growth stimulating effect was shown in nasopharyngeal carcinoma [39], while an oncogenic role was reported for miR-222-3p in diffuse large B-cell lymphoma, by promoting proliferation and inhibiting cell apoptosis [40]. Five constructs had a decreased abundance over time, i.e., pCDH-miR-26a, pCDH-miR-26b, pCDH-miR-150, pCDH-miR-34a, and pCDH-miR-34c. In line with our results, the miR-34 family was shown to function as tumor suppressors in numerous cancer types [8]. However, the miR-34 family members were not expressed in the BL cell lines or normal GC-B cells, which raises the question how relevant these data are in the context of BL pathogenesis or GC B-cell functionality. The other three miRNAs, i.e., miR-150-5p, miR-26a-5p, and miR-26b-5p, were all expressed at moderate to high levels in GC-B cells (RPM > 1000) and have decreased expression in BL cell lines. The negative effect of miR-150-5p on BL cells was consistent with our previous study, in which we showed that targeting of ZDHHC11/B and MYB by miR-150 contributes to the observed phenotype [16]. The miR-26 family has emerged as a key tumor suppressor that is deregulated in many cancer types and plays a regulatory role in cell proliferation, cell cycle, and apoptosis in many types of cancer [41]. EZH2 is a proven target of miR-26a/b-5p and it was shown that MYC could enhance the EZH2 levels by repressing miR-26a/b-5p [42]. Moreover, EZH2 was also identified as an essential gene for BL in our CRISPR/Cas9 dropout screen. In addition, EZH2 was shown to promote MYC expression by inhibiting the MYC-targeting miR-494-3p, resulting in a positive feedback loop between MYC, miR-26a/b-5p, EZH2, and miR-494-3p, which ensured high MYC levels and proliferation of BL [17,42]. A recently published study also confirmed the interplay between MYC, miR-494-3p, and EZH2 in BL [43].

Ago2-IP was used to identify the target genes of miR-26b-5p. The top five identified miR-26b-5p target genes included the previously proven targets EZH2 and KPNA2, and in addition revealed COPS2, NOL12, and MRPL15 as potential targets. Luciferase reporter assays for the latter four genes confirmed binding of miR-26b-5p to the predicted target site of KPNA2. For the other three genes, actual binding of miR-26b-5p to the predicted binding sites could not be confirmed, although a mild effect was observed for COPS2. Nevertheless, we cannot rule out binding of miR-26b-5p to other parts of the transcripts lacking the canonical seed sequences. COPS2 and NOL12 were shown to accelerate cell cycle and promote cell proliferation in several types of tumors [44,45,46]. MRPL15 is a member of human mitochondrial ribosomal proteins (MRPs), which provide energy in the form of ATP for cell growth [47]. Although we show an effect of targeting of these three genes on BL growth, the relevance of these three genes as targets of miR-26b-5p in BL needs further investigation.

KPNA2 is a member of the karyopherin family and was reported to be upregulated in many cancer types [48,49,50,51]. Moreover, KPNA2 was previously validated as a target of miR-26b-5p, but was not yet studied in the context of BL [23,24,49]. The miR-26b-5p-induced KPNA2 knockdown inhibited epithelial ovarian carcinoma cell proliferation and metastasis probably via the KPNA2/OCT4 pathway suppression [23]. In gastric cancer, miR-26b-5p inhibited metastasis by regulating the KPNA2/c-jun pathway [49]. In ovarian carcinoma, KPNA2 promoted cell proliferation and G1/S cell cycle transition through increased levels of MYC [52]. In glioma, knockdown of KPNA2 decreased the levels of MYC and this was linked to decreased proliferation and invasion [53]. The above studies suggest that the miR-26b-5p-dependent regulation of KPNA2 might affect tumor cell growth via diverse mechanisms, including regulation of OCT4, c-jun, and MYC. Altogether, decreased miR-26b-5p levels might be relevant for BL pathogenesis, to ensure high levels of the two target genes, i.e., EZH2 and KPNA2, both of which support MYC activity. BL cells strongly depend on high MYC levels, and downregulation of MYC via the miR-26b-5p/KPNA2 and miR-26b-5p/EZH2 axis could exert negative effect on BL cells. Together with the MYC-dependent repression of miR-26 that we confirmed in BL, this establishes a positive feedback loop that enforces high MYC levels and a high proliferation rate in BL cells.

In summary, we identified in a loss- and gain-of-function screening, 18 miRNAs that control BL cell growth. These included miR-26a-5p and miR-26b-5p, which were both downregulated in BL, was repressed by MYC, and inhibited BL cell proliferation. Eight Ago2-IP-enriched miR-26b-5p targets were shown to be essential for the BL cell growth, and we confirmed KPNA2 as a relevant miR-26b-5p target in BL. In combination with the previously reported and validated target EZH2, we proposed that the MYC-dependent repression of miR-26b-5p is essential to induce high levels of both KPNA2 and EZH2, both of which support MYC-dependent growth of BL.

## 4. Materials and Methods

### 4.1. Tissue Samples and Cell Lines

GC-B cells were sorted from tonsil tissues, as described previously, and frozen BL tissue samples were obtained from the pathology files of the UMCG tissue bank [13,54]. The procedures were according to the guidelines of the medical ethics board of the University Medical Center Groningen. Written permissions for the use of the tonsil samples were obtained from the parents of the children [55].

EBV-negative BL cell lines were obtained from American Type Culture Collection (ATCC, Manassas, VA, USA) (ST486 and Ramos), or German Collection of Microorganisms and Cell Culture (DSMZ, Braunschweig, Germany (DG75 and CA46). P493-6 cells were a kind gift from Prof. D. Eick (Helmholtz Center, Munich, Germany). All cells were cultured at 37 °C, under an atmosphere containing 5% CO_2_ in RPMI-1640 medium (Cambrex Biosciences, Walkersville, MD, USA), supplemented with 2 nM ultraglutamine, 100U/mL penicillin, 0.1mg/mL streptomycin, and 10% (DG75, CA46, Ramos, P493-6) or 20% (ST486) Fetal Bovine Serum (Sigma-Aldrich, Zwijndrecht, The Netherlands). We routinely confirmed cell line identity using the PowerPlex^®^ 16HS System (Promega, Leiden, The Netherlands) and absence of mycoplasma contamination.

### 4.2. Generation of the Construct Pools for miRNA Inhibition and Overexpression

The miRNA inhibition library (miRZip pool) included 58 miRNA inhibition constructs and two negative control constructs. Thirty-four miRNAs were selected based on being MYC-induced, as identified in the P493-6 B cell model or on the basis of being upregulated in BL compared to (1) GC-B cells, (2) chronic lymphocytic leukemia (CLL), or (3) other B-cell lymphoma tissue samples (Table S3) [9,10,11,12,13]. The remaining 24 miRNA inhibition constructs were included in the library, based on availability. The miRZip^TM^/pGreen-Puro Lentiviral-based miRNA inhibition/shRNA constructs were partly purchased from SBI (Palo Alto, CA, USA) and partly custom-made [55]. In addition to the miRNA inhibition constructs, the pool included 186 additional shRNA constructs against various coding or noncoding transcripts irrelevant to this project.

The miRNA overexpression library (pCDH pool) included 44 miRNA overexpression constructs and one negative control construct. The miRNA precursor constructs encoded for 88 potential mature miRNAs, i.e., the 5p and the 3p strands. Five of the 3p strands were not annotated in miRBase (http://www.mirbase.org). Of the 88 potential mature miRNAs, 46 miRNAs derived from 35 constructs were identified as MYC-repressed miRNAs, in the P493-6 B-cell model, or showed decreased expression levels in BL, as compared to (1) GC-B cells, or (2) CLL, or (3) other B-cell lymphomas (Table S4) [9,10,11,12,13]. The remaining nine constructs were included, based on availability. The miRNA overexpression constructs were partly purchased from System Biosciences (SBI, Palo Alto, CA) and partly custom made. To avoid differences in efficiency of virus generation and infection, we designed all inserts with a similar size varying from 481 to 525bp, as described previously [56]. For pCDH-miR-19b-1 and pCDH-miR-27a, two copies of the stem-loop fragment were cloned into the vector, to reach a similar insert size as the other constructs.

Sequences of all inserts were verified by Sanger sequencing. For generation of lentiviral particles of miRNA inhibition and overexpression pools, equal amounts of miRZip or pCDH constructs were mixed respectively.

### 4.3. Production of Lentiviral Particles

Lentiviral particles for miRZip/pCDH pools or individual constructs were produced in HEK-293T cells, by calcium phosphate precipitation transfection, using a third-generation packaging system, as described previously [55]. In brief, the HEK-293T cells were seeded in 6-well plates and grown until ~80% confluence. A plasmid mix consisting of 15 μL CaCl2 (2.5M), 1 μg pMSCV-VSV-G, 1 μg pRSV.REV, 1 μg pMDL-gPRRE, 2 μg lentiviral vectors (plasmid mix or plasmids with individual constructs), and 150 μL of 2x HBS was prepared to transfect the HEK-293T cells. Virus was harvested and filtered using a 0.45 μm filter, 48 h after transfection. Virus was either used directly or stored at –80 °C.

### 4.4. Quality Control of miRNA Inhibition and Overexpression Pools

To verify the presence of the functional antisense miRNA strand of the inhibition constructs, ST486 cells were infected with the miRZip pool (including 55/60 miRZip constructs). HEK-293T cells were infected with the pCDH pool (including 39/45 pCDH constructs) to monitor efficiency of the overexpression constructs, as the maximum percentage of the infected cells was relatively low for the pCDH vector in the BL cell lines. Total RNA was extracted from infected cells using the miRNeasy mini kit (Qiagen, Venlo, The Netherlands), following the manufacturer’s instructions. RNA concentration was measured with a NanoDrop^TM^ 1000 Spectrophotometer (Thermo Fisher Scientific Inc., Waltham, Massachusetts, USA) and RNA integrity was checked on a 1% agarose gel. Small RNA libraries were generated from about 1 μg RNA isolated from the miRZip pool-infected ST486, empty vector (pCDH-EV) and pCDH pool-infected HEK-293T cells, using the NEXTflex™ Small RNA Sequencing Kit v3 (Bio Scientific, Austin TX, USA), as described previously [55]. Total read counts per condition were standardized to 1,000,000.

For the miRZip pool infected ST486, reads were aligned to 5p or 3p strand of the insert sequences of the miRZip constructs. Expression of the appropriate strand of the miRZip constructs was determined by analyzing the abundance of the antisense 3p strand, relative to the 5p strand. For the pCDH pool, effectiveness of the overexpression constructs was estimated by the fold change in the expression of the more abundant miRNA strand in the pCDH pool-infected HEK-293T cells, as compared to empty vector-infected HEK-293T cells.

### 4.5. High Throughput Screen

To achieve a good representation of all constructs, 7.5 million ST486 and DG75 cells were infected in duplicate with the miRZip or pCDH pools, aiming at 10%–15% infected GFP+ cells. This resulted in an average of at least ~3000 (miRZip) and ~16,000 (pCDH) infected cells per construct, at the start of the experiment. At day 5 (miRZip) or day 6 (pCDH) post infection, the cells were sorted using a MoFlo sorter with a 70 μm nozzle (BD Biosciences, Dan Jose, California, USA). For each infection, 1 million sorted GFP+ cells were used for DNA isolation and at least 1 million of the sorted cells were kept in culture, until day 40, to ensure sufficient representation of each construct. For the miRZip screens, 1 million cells were collected at days 5, 7, 15, 25, and 40 (DG75) or days 5, 9, 15, 25, and day 40 (ST486). For the pCDH screens, 1 million cells were collected at days 6, 8, 15, 25, and 40 (DG75 and ST486). Cell pellets were stored in −20 °C for DNA isolation.

### 4.6. DNA Isolation and Amplification of the Inserts

Genomic DNA was isolated using a salt/chloroform extraction method and was measured by a NanoDrop^TM^ 1000 Spectrophotometer (Thermo Fisher Scientific Inc.). DNA quality was checked on a 1% agarose gel. Inserts of the constructs were amplified in duplicates (miRZip) or triplicates (pCDH), using ampliTtaq DNA Polymerase, following the instructions of the manufacturer (Thermo Fisher Scientific Inc.). For the miRZip samples, a universal forward primer 5′-CTGGGAAATCACCATAAACG-3′ with a unique 8-9nt sample ID was used for each individual PCR reaction. The sequence of the reverse primer was 5′-CTAACCAGAGAGACCCAGTAG-3′ for ST486 samples and 5′-TCTAACCAGAGAGACCCAGTAG-3′ for DG75 samples. For the pCDH samples, the sequence of the universal forward primer was 5′-CTGGGAAATCACCATAAACG-3′, with a unique 8-9nt sample ID for each individual PCR reaction. The sequence of the reverse primers was 5′-CAAGCGGCTTCGGCCAGTAACGTT-3′ for the ST486 samples and 5′-CCAAGCGGCTTCGGCCAGTAACGTT-3′ for the DG75 samples. Approximately 400 ng genomic DNA, equivalent to about 67,000 cells (~6pg DNA/cell), was used for each PCR reaction. This DNA input corresponded to at least 53,000 or 27,000 GFP+ cells per PCR, for the miRZip (minimal purity 75%) and the pCDH (minimal purity 45%) samples, respectively. PCR products were checked on a 2% agarose gel and mixed equally, based on band intensities.

### 4.7. Library Preparation, Next Generation Sequencing, and Data Analysis

The PCR product mixes of the miRZip and pCDH pools were purified using the DNA Clean & Concentrator^TM^-5 kit (Zymo Research, Irvine, CA, USA). For the miRZip constructs, this was followed by digestion with AgsI (SibEnzyme, Academtown, Russia), which cuts within the loop sequence of the short hairpins, to prevent formation of unwanted hairpin-like structures during the sequencing reaction. Adaptors (NEBNext multiplex oligo’s for Illumina, #E7335 New England Biolabs, Ipswich, Massachusetts, USA) were ligated to the purified DNA fragments, followed by paired-end sequencing, using the MiSeq^TM^ platform (Illumina, San Diego, CA, USA). The sequencing reads were assigned to the PCR samples, using the sample IDs and were aligned to the insert sequences of the constructs in the pools. Processing of the reads and alignment was done using SAM tools (version 1.3; http://www.htslib.org) and BWA (version 0.7.12; https://github.com/lh3/bwa), respectively. The total reads of the miRZip samples were normalized to 50,000 and the total reads of the pCDH samples were normalized to 20,000, based on the estimated number of cells present in each PCR reaction.

Processing of the high-throughput screening data was performed, as described previously [57]. In brief, fold changes of reads per construct relative to day 5 (miRZip) or day 6 (pCDH) were determined for each independent infection, based on the average reads of replicate PCRs. For the constructs showing increased fold changes, the calculated fold changes were corrected by subtracting 1 from each value and for the constructs with decreased fold changes, the calculated fold changes were corrected by adding up 1. The adapted fold changes of all time points for a construct were plotted and the slope of the resulting trend line with the starting point forced to 0 was determined using a commercial software package (MATLAB 6.1, The MathWorks Inc., Natick, MA, USA, 2000). An adapted Tukey interquartile (IQR) method with a lower band cutoff of Q1-(1xIQR) and an upper band cutoff of Q3+(1xIQR) was applied to all slopes, to identify the constructs with significantly altered abundance in the cell populations. Based on the adapted IQR method analysis, consistently increased or decreased abundance in two infections of at least one BL cell line was set as the minimal criterion of altered abundance for each construct.

### 4.8. Green Fluorescent Protein (GFP) Growth Competition Assay

To validate the results of the high-throughput screens, BL cell lines were infected by individual miRZip, pCDH, or CRISPR/Cas9 constructs, aiming at an infection efficiency of 20% to 50% GFP+ cells on day 4 or day 6. The percentage of GFP+ cells was monitored by flow cytometry (BD Biosciences, San Jose, CA, USA), for a period of 22 days. GFP+ percentages were normalized to the percentage of GFP+ cells at day 4 (miRZip) or day 6 (pCDH and CRISPR/Cas9). Statistical analysis of GFP competition assays was performed, as described previously [55]. In brief, decrease in percentages of GFP+ cells over time was compared with the controls, using a mixed model, with time and the interaction of time and construct types as fixed effects and the measurement repeat within construct types as random effect in SPSS (22.0.0.0 version, IBM, Armonk, New York, USA).

### 4.9. MiRNA qRT-PCR

The miRNeasy mini- or microkits (Qiagen) were used to isolate RNA, according to the manufacturer’s instructions. RNA concentrations were measured by a NanoDrop^TM^ 1000 Spectrophotometer (Thermo Fisher Scientific Inc.) and RNA integrity was evaluated on a 1% agarose gel. Expression of miR-26a-5p and miR-26b-5p was analyzed using Taqman miRNA quantitative PCR assays (Thermo Fisher Scientific Inc.), in a multiplexed fashion, as described previously [58]. Taqman assays were designed to detect 5′-UUCAAGUAAUCCAGGAUAGGC-3′ (Cat no.: 00405) for miR-26a-5p and 5′-UUCAAGUAAUUCAGGAUAGGU-3′ (Cat no.: 00406) for miR-26b-5p. MiRNA expression levels were normalized to RNU49 (house-keeping gene). Cycle crossing point (Cp) values were determined with Light Cycler 480 software version 1.5.0 (Roche, Basel, Switzerland). Relative expression levels of miRNAs were determined by calculating 2−∆Cp (∆Cp = CpmiRNA − CpRNU49).

### 4.10. Ago2-RIP-Chip

Six million ST486 or DG75 cells were infected with pCDH-miR-26b construct or pCDH-EV. Approximately 35 million cells were harvested either directly or after sorting, to reach a GFP+ percentage >80% for each condition on day 9 (ST486) or day 11 (DG75), post infection. Immunoprecipitation (IP) of the Ago2-containing RISC was performed, as described previously [59]. For each condition, about 50 ng RNA of total (T) and Ago2-IP (IP) fractions were labeled and hybridized to a commercially available Agilent microarray (AMADID no.: 072363). Data analysis was performed as described previously [55]. In brief, the probes flagged as present in all total fractions of pCDH-miR-26b or pCDH-EV infected samples with expression levels in the 25th to 100th percentile were included in the downstream analysis. IP/T ratios were calculated for all probes showing a consistent signal in the duplicate experiments. Probes with an IP/T ratio ≥2 were considered as potential miRNA targets and the probes showing ≥2-fold increase in the IP/T ratio of the miR-26b overexpressing cells, as compared to the IP/T ratio of empty vector (pCDH-EV) were considered to be potential miR-26b-5p targets.

### 4.11. Prediction of MiRNA Binding Sites and Gene set Enrichment Analysis

To identify putative targets of miR-26b-5p among the Ago2-IP enriched genes, we analyzed the presence of binding sites using TargetScan release 7.2 (http://www.targetscan.org) [60] in genes enriched in the Ago2-IP and in genes expressed in the total fraction. Significance of the enrichment in the Ago2-IP was determined using the goodness of fit chi-squared test. In addition, we used a Pearl script to identify the miR-26b-5p binding sites (7mer-A1, 7mer-m8, and 8mer) in the 5′-UTR, CDS, and 3′-UTR of the experimentally identified targets of miR-26b-5p, based on the RefSeq transcripts. For transcripts without an Ensembl ID, i.e., lncRNA genes, we performed a manual search for potential miR-26b-5p binding sites, using the LNCipedia or TCONS-transcript ID.

Gene set enrichment analysis on the ranked miR-26b-IP/T ratio over the EV-IP/T ratio values was performed for the 5223 gene sets (Sets H, C2, C3-miR and C6), from the Molecular Signatures Database V6.2 (http://software.broadinstitute.org/gsea/msigdb). The C3-miR gene sets included a miR-26a/b-5p set (TACTTGA_MIR26A_MIR26B) containing 301 predicted miR-26a/b-5p target genes. We also included a miR-26b-5p predicted target gene set including the top-500 TargetScan-predicted target genes ranked by the cumulative weighted context++ score). The overlap between the list of 301 and 500 predicted miR-26b-5p target genes was 125 genes.

### 4.12. Genome-Wide CRISPR/Cas9 Knockdown Screen for Essential Genes in BL

To facilitate selection of candidate miR-26b-5p target genes relevant for BL cell growth, we used data from our genome-wide CRISPR/Cas9 knockout screen in the ST486 cells, using the Brunello library (Addgene, #73179) in which each gene is targeted by four single guide (sg)RNAs [28]. In brief, 130 million ST486 cells were infected in duplicate to achieve ~30% infected cells and an average of 500x coverage per sgRNA in the library. The transduced cells were selected with puromycin (0.3 µg/mL) for four days, starting 24 h after transduction. After puromycin selection, at least 38 million cells (corresponding to 500× coverage of the library) were collected (T0) and the remaining cells were harvested after culture for 20 population doublings, maintaining the 500× coverage at each passage (T1). DNA was isolated and sgRNA inserts were amplified in 130 PCR reactions per sample (3 µg DNA per 50 µl reaction) using primers containing Illumina adaptors and sample-specific barcodes, as described previously [61]. Amplicons were mixed, based on band intensities, purified from gel and subjected to NGS on the Illumina X-Ten platform (BGI, Hong-Kong, China). Reads were aligned to the sgRNA constructs in the library and enumerated using a Python script [61]. To identify genes significantly depleted or enriched from the cell pool (p_adj_ < 0.001), the DeSeq2 algorithm was applied using the CRISPRAnalyzeR tool (http://crispr-analyzer.dkfz.de). For validation, the two most significantly depleted sgRNAs were ordered and cloned into the lentiCRISPR v2 vector with GFP co-expression, for the GFP growth competition assay (Table S5). The modified lentiCRISPR v2 vector was a kind gift from Ryan O’Connell of the University of Utah (Salt Lake City, UT, USA).

### 4.13. Validation of MiR-26b-5p Binding to the Predicted Binding Sites

MiR-26b-5p binding sites (wild type and mutated) of the four selected target genes (Table S6) were cloned between the Xhol and NotI restriction sites of the psi-Check-2 vector (Promega, Madison, WI, USA) downstream of the *Renilla* luciferase reporter gene, driven by the SV40 promoter. This vector also contained the firefly luciferase gene expressed from the TK promoter, which was used for normalization. Luciferase reporter assay was performed, as described previously [30]. In briefl, the psi-Check-2 vectors with WT or mutated miR-26b-5p binding sites were co-transfected with either 10 µM pre-miR-26b (Cat. NO.: AM17100) or control oligos (Cat. NO.: AM17111) to ST486 cells, using an Amaxa nucleofector device (program A23) and the Amaxa Cell Line Nucleofector Kit V (Cat NO.: VACA-1003) (Amaxa, Gaithersburg, MD, USA). Cells were harvested 24 h after transfection. *Renilla* and firefly luciferase activities were measured in the cell lysate using a Dual-Luciferase Reporter Assay System (Promega). Each experimental condition was measured in duplicate and the results were averaged. For each construct, the luciferase assay was performed in three independent biological replicates.

## 5. Conclusions

MicroRNAs are important regulators of the Burkitt lymphoma pathogenesis. Our high-throughput based miRNA loss- and gain-of-function screen identified 18 miRNAs that influence BL cell growth. Further analysis of the MYC-repressed miR-26b-5p confirmed its negative effect on BL cell growth and identified KPNA2 and EZH2 as miR-26b-5p targets that are essential genes in BL. Both KPNA2 and EZH2 are known to promote MYC expression. This suggests a feedback loop in BL, where miR-26b-5p is repressed by MYC to release its negative effect on KPNA2 and EZH2, and further promotes elevated MYC levels and a high proliferation rate.

## Figures and Tables

**Table 1:** Number of Ago2-IP enriched genes in the miR-26 overexpressing and control BL cell lines, and the number of miR-26b-5p target genes in ST486 and DG75.

**IP/T Ratio**	**ST486 (*n* = 12,286)**			**DG75 (*n* = 9206)**		
	**pCDH-miR-26b**	**EV**	**pCDH-miR-26b/EV**	**pCDH-miR-26b**	**EV**	**pCDH-miR-26b/EV**
≥2	1587	1524	94	1236	1140	59
≥4	560	573	10	462	412	5
≥8	169	205	0	141	143	0

**Table 2:** Overview of the Ago2-IP and Brunello results for the 47 miR-26b-5p targeting genes.

Gene	Transcript ID	FC of IP/T Ratio		Brunello Screen		miR-26b-5p Binding Site **	
		DG75	ST486	FC	*p*-Value *	CDS	3′UTR
COPS2	ENST00000388901	5.1	4.0	−13.9	4.97 × 10^−86^	7mA1	7mA1
EZH2	ENST00000320356	2.3	2.2	−9.4	3.87 × 10^−45^	7mA1	8m
KPNA2	ENST00000330459	2.5	3.3	−6.6	1.16 × 10^−6^		8m
MRPL15	ENST00000260102	2.2	2.7	−6	5.03 × 10^−43^	7mA1	
NOL12	ENST00000359114	2.2	2.1	−5.6	2.67 × 10^−47^	8m	7mA1
EIF4E	ENST00000505992	2.9	4.3	−4.3	5.76 × 10^−12^		
TRAPPC4	ENST00000533632	2.1	2.3	−3.7	1.0 × 10^−8^		7mA1
ALG1	ENST00000262374	2.3	2.5	−3.2	0.042		7m8
FANCF	ENST00000327470	3.4	2.6	−2.4	3.52 × 10^−4^		7mA1/8m
B3GNT2	ENST00000301998	3.1	2.9	−1.9	1	8m	
MT2A	ENST00000245185	3.4	2.4	−1.7	0.045		
NXT1	ENST00000254998	2.3	2.3	−1.5	1		
MT1B	ENST00000334346	2.3	2.2	−1.4	1		
MSMO1	ENST00000261507	2.3	2.5	−1.4	1		8m
PPP1CC	ENST00000335007	2.9	3.8	−1.2	1	7mA1	
MT1E	ENST00000306061	2.2	2.7	−1.2	1		
MPV17L2	ENST00000599612	2.6	2.5	−1.2	1	8m	
RHOQ	ENST00000238738	2.8	2.5	−1.2	1		7mA1/7m8/8m
TBC1D7	ENST00000379300	2.0	2.8	−1.2	1	8m	
TMEM156	ENST00000381938	3.0	2.8	−1.1	1	8m	7mA1
REEP4	ENST00000306306	4.6	4.1	−1.1	1		7m8
ASB10	ENST00000420175	2.1	3.4	−1.1	1		
OSCP1	ENST00000235532	2.9	2.4	-1	1		
H3F3C	ENST00000340398	2.3	3.0	-1	1		
PRMT3	ENST00000331079	2.1	2.7	1	1	7mA1	
ZDHHC6	ENST00000369405	4.2	3.9	1	1		8m
DIABLO	ENST00000650715	2.1	2.1	1.1	1		7mA1
TXNDC17	ENST00000250101	2.5	2.1	1.1	1	7mA1	
MGST1	ENST00000396209	3.5	4.5	1.1	1		
MSRB2	ENST00000376510	3.8	3.8	1.2	1		7mA1
FRAT2	ENST00000371019	3.7	3.3	1.2	1		8m
ADAM19	ENST00000257527	2.4	2.1	1.3	1		8m
PRKCD	ENST00000330452	4.2	3.1	1.3	1		8m
ACBD5	ENST00000396271	3.3	2.5	1.3	1		7mA1/8m
ZNF410	ENST00000555044	2.4	2.4	1.3	1		8m
ACYP2	ENST00000394666	2.4	2.6	1.4	1		
BID	ENST00000317361	2.7	3.6	1.6	1		8m
SLC25A36	ENST00000324194	2.3	2.3	1.6	1		
CRADD	ENST00000332896	2.0	2.4	1.6	1		8m
POLR3G	ENST00000369314	2.4	3.9	1.7	1		
SAAL1	ENST00000524803	2.2	2.3	1.7	0.984	7mA1/8m	
TMEM206	ENST00000261455	3.0	4.2	1.8	6.96 × 10^−4^		
LINC00847	ENST00000501855	2.8	2.9				
LOC100294145	N/A	2.2	2.1				
MT1L	ENST00000565768	2.8	2.3				
XLOC_l2_008009	TCONS_l2_00014564	2.5	2.9				
lnc-C2orf81-2	lnc-C2orf81-2:1–2	2.3	2.2				7m8/8m ***

* Cut-off for the significantly depleted or enriched genes: adjusted *p*-value < 0.001. ** No miR-26b-5p binding sites were present in 5′-UTR. *** Binding sites in noncoding RNAs were listed in the 3′-UTR column. 7mA1 = 7mer-A1, 7m8 = 7mer-m8, 8m = 8mer, FC = fold change, and N/A = not available.

## Supplementary Materials

The following are available online at https://www.mdpi.com/2072-6694/12/6/1464/s1. Figure S1: Quality control of miRNA inhibition and overexpression pools, Figure S2: Sorting of the infected BL cells, agarose gel electrophoresis of the PCR products, and sequencing reads overview of the miRZip and pCDH pools, Figure S3: Ago2-IP experiment upon miR-26b-5p overexpression, Figure S4: Uncropped western blot results for the Ago2 protein in Total fraction (T), flow through fraction (FT), and Ago2-immunoprecipitation fraction (IP) samples, Figure S5: Volcano plot visualizing the results of the Brunello screen in ST486, Table S1: An overview of the miRZip pool infected samples and NGS raw read counts, Table S2: An overview of the pCDH pool infected samples and the NGS raw read counts, Table S3: Overview of the 58 miRNAs included in the miRZip pool, Table S4: Overview of the 44 miRNA precursors included in the pCDH pool, Table S5: Oligos sequences of sgRNAs. Table S6: Oligos for cloning miR-26b-5p binding sites from selected genes.
