# Supplementary material for: The miR-26b-5p/KPNA2 Axis Is an Important Regulator of Burkitt Lymphoma Cell Growth
Source: Cancers (Basel). 2020 Jun 4;12(6):1464. doi: 10.3390/cancers12061464 (PMC7352454; doi:10.3390/cancers12061464)
Supplement: Supplementary file 1 [file cancers-12-01464-s001.pdf]

cancers

MDPI

Supplementary Materials

The miR-26b-5p/KPNA2 Axis Is an Important Regulator of Burkitt Lymphoma Cell Growth

Fubiao Niu, Marta Kazimierska, Ilja M. Nolte, Miente Martijn Terpstra, Debora de Jong, Jasper Koerts, Tineke van der Sluis, Bea Rutgers, Ryan M. O'Connell, Klaas Kok, Anke van den Berg, Agnieszka Dzikiewicz-Krawczyk and Joost Kluiver

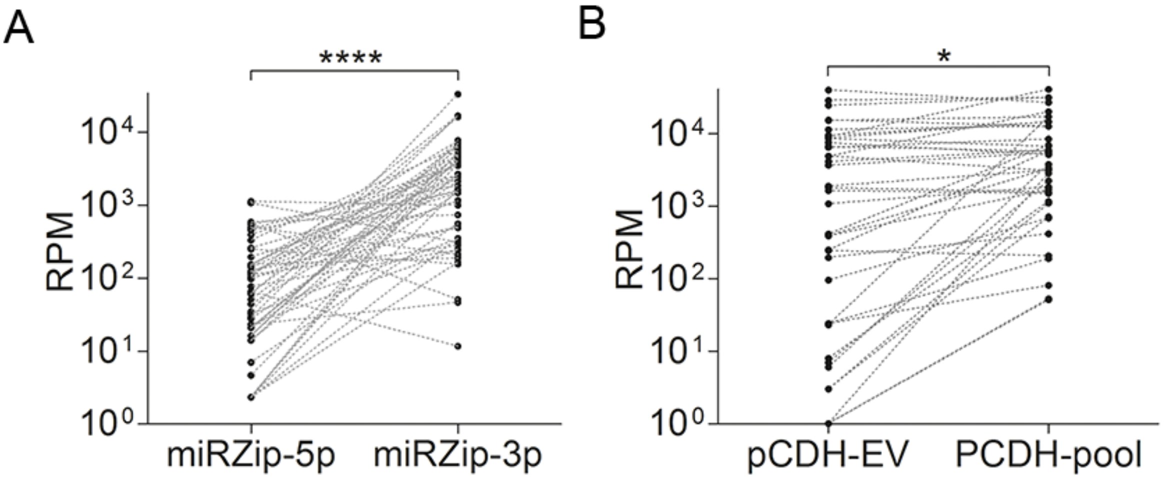

Figure S1. Quality control of miRNA inhibition and overexpression pools. (**A**) Read counts of the sense (miRZip-5p) and antisense (miRZip-3p, functional strand) strands of the miRZip constructs in ST486 cells infected with the miRNA inhibition library (55/60 constructs). Cells were harvested on day 6 post infection for RNA isolation and library preparation. Almost 16 million reads were generated. (**B**) Expression of mature miRNAs in HEK-293T cells transduced with the miRNA overexpression library (pCDH-pool, 39/45 constructs) compared to empty vector (pCDH-EV) transduced cells. Cells were harvested on day 6 post infection. Approximately 13 and 16 million reads were obtained from the pCDH-pool and pCDH-EV infected cells, respectively. RPM = reads per million. *$p < 0.05$, ****$p < 0.0001$ (Paired *t*-test).

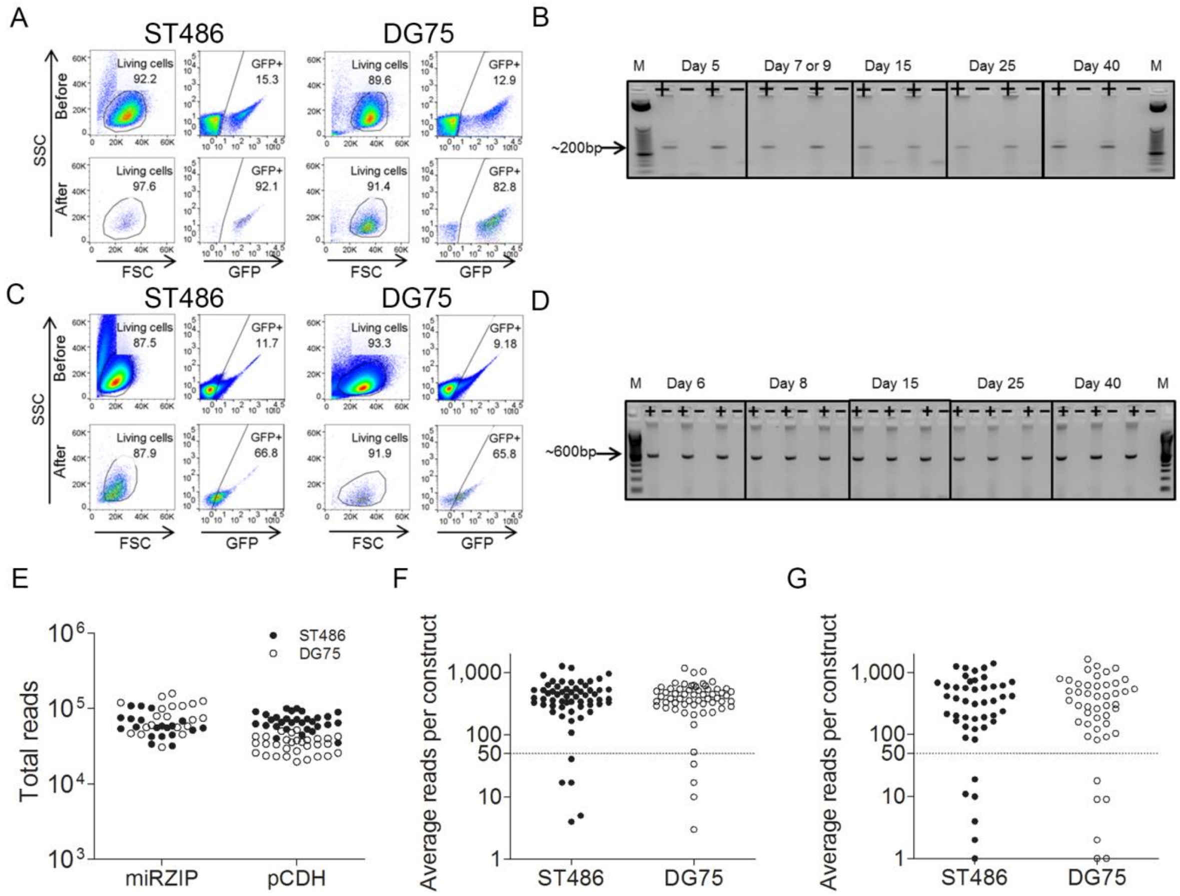

Figure S2. Sorting of infected BL cells, agarose gel electrophoresis of the PCR products, and sequencing reads overview of miRZip and pCDH pools. GFP+ cells were sorted at day 5 for (**A**) miRZip pool infected samples and day 6 for (**C**) pCDH pool infected samples. (**B**) Sizes of miRZip PCR products range from 172 to 194 bp. (**D**) Sizes of pCDH PCR products range from 572 to 602 bp. +, PCR reaction with genomic DNA; -, no template control; M, 25 bp or 100bp molecular weight DNA ladder. (**E**) Total reads of 40 miRZip and 60 pCDH samples. Average read counts per construct in (**F**) miRZip and (**G**) pCDH samples on sorting day. Constructs with a minimum of 50 reads in both BL cell lines were used for further analysis.

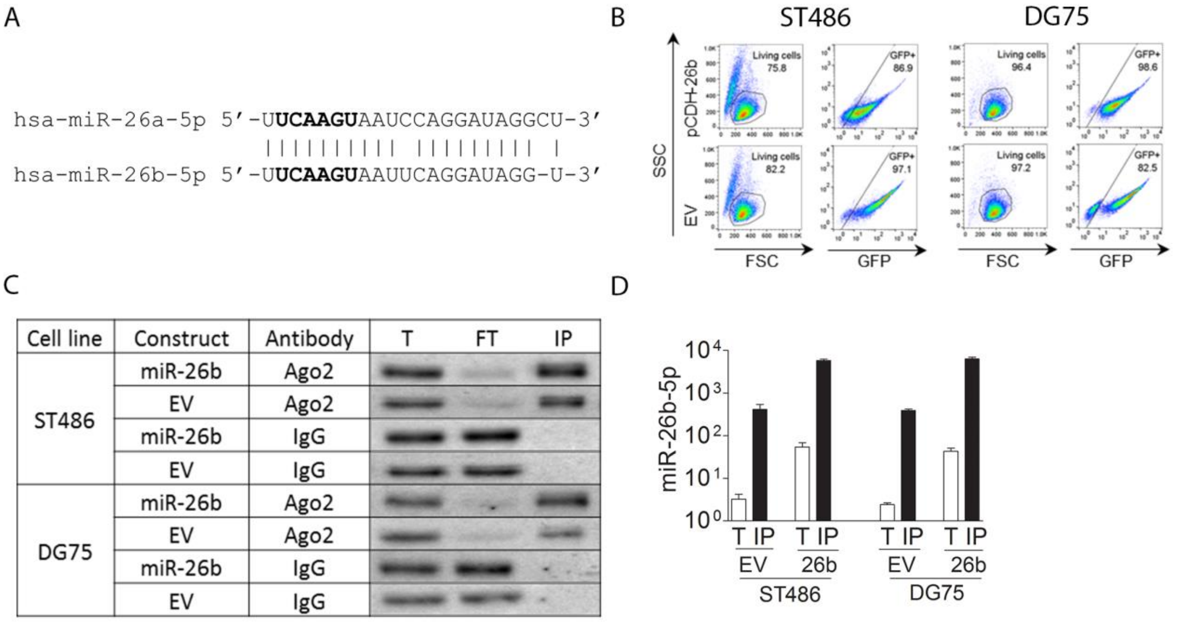

Figure S3. Ago2-IP experiment upon miR-26b-5p overexpression. (**A**) Sequences and alignment of miR-26a-5p and miR-26b-5p. The nucleotides in bold represent seed region. (**B**) Infection of ST486 and DG75 cells with miR-26b overexpression and negative control constructs. Cells were harvested at day 9 (ST486) and day 11 (DG75) post infection. (**C**) Western blot of Ago2 protein in total (T), flow through (FT), and immunoprecipitated (IP) fractions after Ago2 or control IgG IP shows Ago2 enrichment in the Ago2-IP fraction but not in the IgG-IP control fraction. (**D**) Overexpression efficiency and enrichment of miR-26b-5p in the IP fraction. MiR-26b-5p levels were 16.8- and 17.9-fold upregulated in ST486 and DG75, respectively, indicating an effective overexpression. In line with the western blot, miRNA qRT-PCR showed strong enrichment of miR-26b-5p in the Ago2-IP fractions, further indicating effective pull down of the miRNA-containing Ago2-RISC complexes.

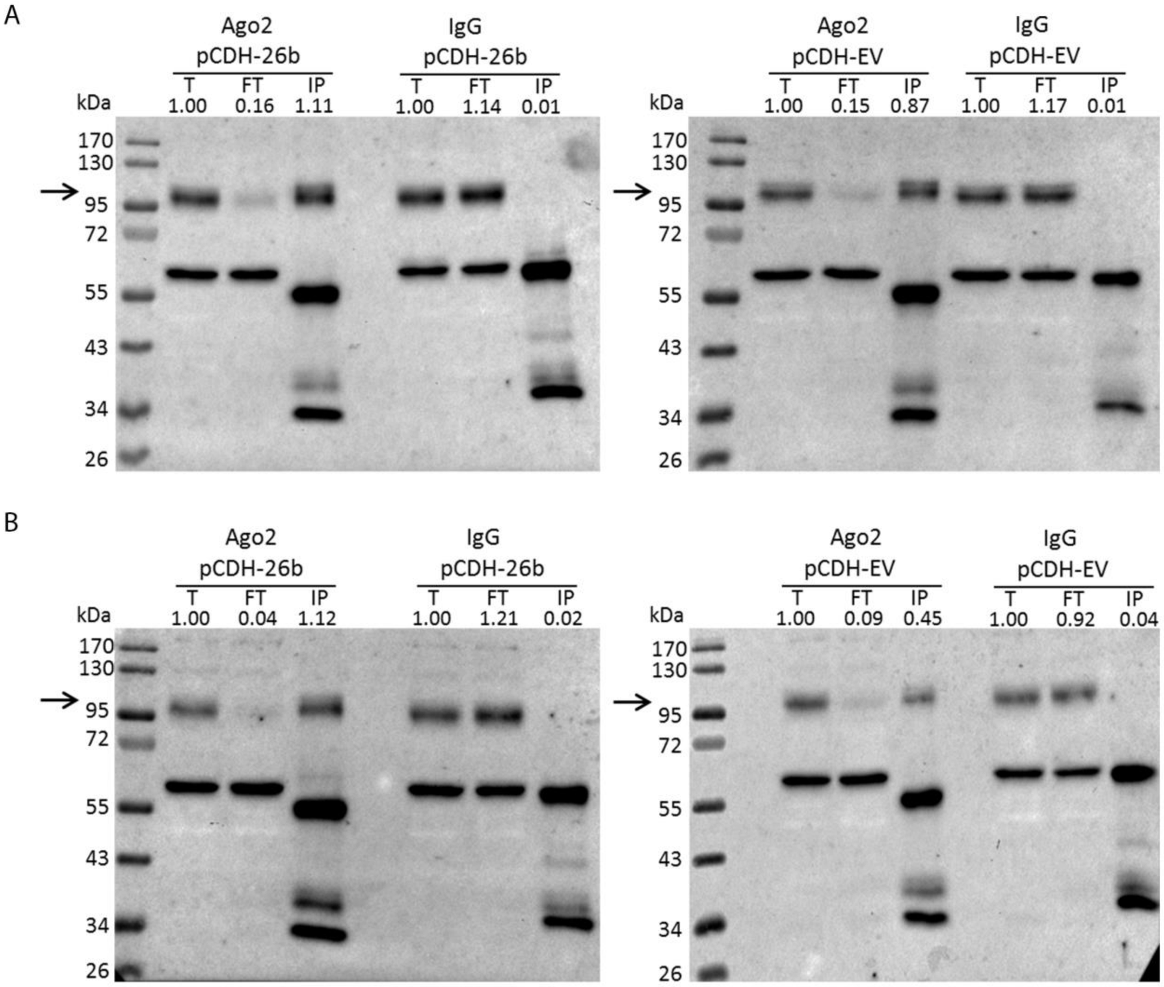

Figure S4. Uncropped western blot results for Ago2 protein in Total fraction (T), flow through fraction (FT), and Ago2-immunoprecipitation fraction (IP) samples using the anti-Ago2 antibody or the control anti-IgG antibody for miR-26b overexpression and the negative control (pCDH-EV) in (**A**) ST486 and (**B**) DG75 (see also Figure S3C). Arrows indicate the AGO2 protein. The T and FT lanes contain an aspecific band at ~60 kDa and the IP lanes also contain bands for the heavy and light chain of the antibody used for IP. For each IP experiment the total fraction is set to 1.

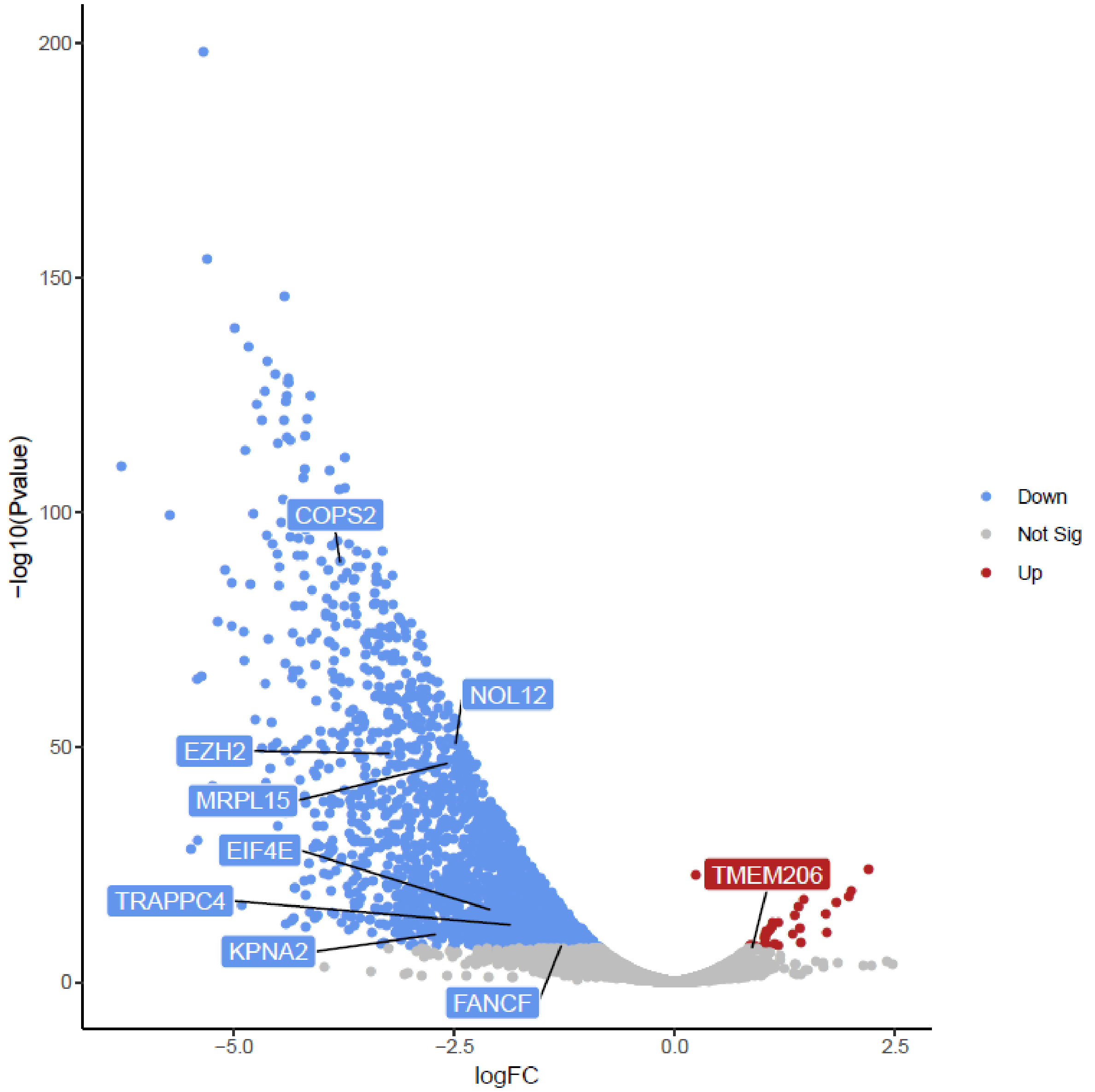

Figure S5. Volcano plot visualizing the results of the Brunello screen in ST486. The gene names indicated in the figure were identified as targets of miR-26b-5p by the Ago2-IP that showed significant changes in abundance in the Brunello screen.

Table S1. An overview of the miRZip pool infected samples and NGS raw read counts.

ST486 1st Experiment	GFP+ (%)	Mapped Read Counts and Percentage	
		PCR 1 (%)	PCR 2 (%)
**5**	93	31,795 (92)	56,673 (92)
9	93	108,732 (93)	101,591 (93)
15	93	51,459 (92)	44,457 (92)
25	92	58,474 (92)	70,032 (92)
40	92	68,249 (92)	53,461 (92)
**ST486 2nd Experiment**			
5	92	107,316 (92)	75,083 (93)
9	88	58,643 (93)	55,291 (92)
15	90	55,313 (92)	42,473 (92)
25	89	33,564 (91)	42,457 (92)
40	89	72,573 (91)	54,945 (91)
**DG75 1st Experiment**			
5	83	157,866 (92)	125,162 (92)
7	82	56,140 (92)	45,032 (93)
15	79	79,209 (92)	119,128 (92)
25	77	98,804 (92)	108,243 (92)
40	75	106,658 (93)	58,098 (92)
**DG75 2nd Experiment**			
5	82	143,826 (93)	115,756 (92)
7	82	78,086 (93)	59,933 (93)
15	80	70,552 (92)	74,650 (93)
25	78	45,146 (92)	56,168 (92)
40	77	46,606 (92)	30,684 (92)

Table S2. An overview of the pCDH pool infected samples and NGS raw read counts.

ST486 1st Experiment	GFP+ (%)	Mapped Read Counts and Percentage		
		PCR 1 (%)	PCR 2 (%)	PCR 3 (%)
**6**	63	71,329 (97)	65,326 (98)	70,674 (98)
8	68	62,557 (98)	40,014 (98)	49,125 (96)
15	56	57,815 (96)	76,006 (98)	60,922 (97)
25	48	52,953 (98)	64,646 (99)	57,869 (98)
40	47	89,435 (96)	93,227 (97)	72,239 (97)
**ST486 2nd Experiment**				
6	57	72,609 (97)	99,881 (98)	91,069 (97)
8	62	99,923 (97)	91,916 (97)	83,151 (97)
15	53	59,275 (94)	64,382 (94)	64,716 (98)
25	45	75,033 (97)	77,715 (99)	66,511 (99)
40	46	52,951 (99)	44,555 (97)	34,878 (99)
**DG75 1st Experiment**				
6	64	34,832 (98)	42,598 (98)	51,590 (98)
8	73	35,737 (98)	38,406 (97)	35,330 (98)
15	72	38,055 (98)	33,745 (95)	33,306 (98)
25	66	47,685 (98)	50,028 (99)	42,360 (99)
40	62	37,446 (99)	27,186 (99)	29,223 (95)
**DG75 2nd Experiment**				
6	62	34,044 (94)	39,928 (95)	39,546 (92)
8	79	23,505 (97)	42,419 (87)	33,133 (98)
15	78	22,791 (98)	31,935 (98)	25,679 (93)
25	72	25,839 (96)	19,569 (70)	20,839 (79)
40	69	23,144 (98)	22,828 (90)	23,210 (97)

Table S3. Overview of the 58 miRNAs included in the miRZip pool.

Construct	Top 30 in BL [19]	Increased in BL vs. GC-B [19]	MYC-Induced in P493-6 [12]	Increased in BL vs. CLL [12]	Increased in eBL vs. GC-B [11]	Increased in BL vs. DLBCL [10]	Increased in BL vs. DLBCL-FL [9]	Increased in BL vs. other Lymphomas [8]	Depleted in Screen
miRZip-let-7b-5p									
miRZip-let-7e-5p									√
miRZip-let-7f-2-3p		√							√
miRZip-9-5p						√			√
miRZip-15a-5p									
miRZip-16-5p	√								
miRZip-17-5p		√	√	√					
miRZip-18a-5p			√	√				√	
miRZip-19a-3p			√	√			√	√	
miRZip-19b-3p	√		√		√			√	
miRZip-20a-5p	√	√	√						
miRZip-21-3p					√				
miRZip-21-5p	√								√
miRZip-23a-3p									
miRZip-23b-3p									
miRZip-24-3p									
miRZip-25-3p	√								
miRZip-26a-5p	√				√				
miRZip-27a-3p									
miRZip-27b-3p					√				
miRZip-29a-3p									
miRZip-30e-5p	√								√
miRZip-34a-5p									
miRZip-92-3p	√		√		√	√		√	
miRZip-99b-5p					√				
miRZip-100-5p									
miRZip-106a-5p			√	√					
miRZip-106b-5p							√		√
miRZip-125a-5p									
miRZip-142-3p									
miRZip-142-5p	√								
miRZip-144-3p									
miRZip-146a-5p	√								
miRZip-146b-5p					√				

miRZip-150-5p							
miRZip-155-5p							
miRZip-181a-5p	√		√				√
miRZip-182-5p	√	√			√		
miRZip-183-5p	√	√			√	√	
miRZip-190a-5p		√					√
miRZip-196a-5p							
miRZip-205-5p							
miRZip-301b							
miRZip-320a		√					
miRZip-324-3p							
miRZip-330-5p		√					
miRZip-345-5p							
miRZip-378a-3p	√	√		√			
miRZip-431-star							
miRZip-449a-5p							√
miRZip-494-3p				√			√
miRZip-500a-5p							
miRZip-615-3p						√	
miRZip-615-3p							
miRZip-625-5p		√					
miRZip-892b							
miRZip-4454		√					
miRZip-4455							

Table S4. Overview of the 44 miRNA precursors included in the pCDH pool.

pCDH Construct	Strand	Decreased in BL vs. GC-B [19]	MYC-Repressed [12]	Decreased in BL vs. CLL [12]	Decreased in eBL vs. GC-B [11]	Decreased in BL vs. DLBCL [10]	Decreased in BL vs. DLBCL-FL [9]	Decreased in BL vs. other Lymphomas [8]	Depleted/Enriched in Screens
let-7a	5p		√	√	√			√	
miR-9	5p					√			
	3p					√			
miR-10a	5p							√	
miR-19b-1									
miR-21	5p	√		√	√				
	3p	√							
miR-23a	5p					√			
	3p		√	√					
miR-24	3p		√	√					
miR-26a	5p		√	√		√		√	√
miR-26b	5p		√	√		√		√	√
miR-27a	3p			√					
miR-27b	3p			√					
miR-28	5p	√		√	√				
	3p	√							
miR-29a	3p		√	√	√			√	
miR-29b	3p		√	√		√		√	
miR-29c	5p						√		
	3p		√	√	√			√	
miR-30a	5p	√		√		√			
	3p	√		√					
miR-34a	5p		√	√					√
miR-34b	5p			√		√			
	3p					√			
miR-34c	5p								√
miR-101	3p		√	√					
miR-106b									
miR-125a	5p	√		√					
miR-125b	5p		√	√		√			
miR-141	3p	√		√					
miR-142	5p			√		√			
	3p		√	√					
miR-145	5p			√					

miR-146a	5p		√	√		√		√	
miR-148	3p		√	√					
miR-150	5p	√	√	√			√	√	√
	3p	√					√		√
miR-151a	5p	√							√
	3p	√							√
miR-155	5p	√	√	√	√	√	√	√	√
miR-181a-2									
miR-181b-2									
miR-200a									
miR-200c	3p	√		√					
miR-205									
miR-221	5p	√							
	3p	√		√	√	√	√		
miR-222	3p	√	√	√	√	√			√
miR-363	3p	√						√	
miR-429									
miR-449a									
miR-451			√			√		√	
miR-486	5p	√							
miR-577									

Table S5. Sequences of the single guide RNAs.

NO.	sgRNA	Sense (S)/ Anti-Sense (AS)	5′ to 3′
1	COPS2_sg1	S	CACCGGTGGTGGTAAAATGCACTTG
		AS	AAACCAAGTGCATTTTACCACCACC
2	COPS2_sg2	S	CACCGCCAGTTACATCAGTCGTGCC
		AS	AAACGGCACGACTGATGTAACTGGC
3	KPNA2_sg1	S	CACCGCGGATTATGTTGTCTATGGG
		AS	AAACCCCATAGACAACATAATCCGC
4	KPNA2_sg2	S	CACCGAAATGAACGAATTGGCATGG
		AS	AAACCCATGCCAATTCGTTCATTTC
5	MRPL15_sg1	S	CACCGAAGTTGGCCAGGCTCACACG
		AS	AAACCGTGTGAGCCTGGCCAACTTC
6	MRPL15_sg2	S	CACCGGTTAAGTCAATAGGTTGACT
		AS	AAACAGTCAACCTATTGACTTAACC
7	NOL12_sg1	S	CACCGGCTCGTTCTTAGCTTCGACG
		AS	AAACCGTCGAAGCTAAGAACGAGCC
8	NOL12_sg2	S	CACCGACCTGACAGGCTTCCACAAG
		AS	AAACCTTGTGGAAGCCTGTCAGGTC
9	NTCR1	S	CACCGACGGAGGCTAAGCGTCGCAA
		AS	AAACTTGCGACGCTTAGCCTCCGTC
10	NTCR2	S	CACCGATCGTTTCCGCTTAACGGCG
		AS	AAACCGCCGTTAAGCGGAAACGATC

Table 6. Sequences of the miR-26b-5p binding sites from selected genes cloned in the reporter vector.

miR-26b-5p Binding Site (MBS)		Sense(S)/ Anti-Sence (AS)	5′ to 3′
COPS2_MBS1	WT	S	**TCGA**TTACCTGCAGAAATTCTCTCACAAATAACCTGCAATAACTTGAAATGCATACCC
		AS	**GGCC**GGGTATGCATTTCAAGTTATTGCAGGTTATTTGTGAGAGAATTTCTGCAGGTAA
	MUT	S	**TCGA**TTACCTGCAGAAATTCTCTCACAAATAACCTGCAATAAGTAGTAATGCATACCC
		AS	**GGCC**GGGTATGCATTACTACTTATTGCAGGTTATTTGTGAGAGAATTTCTGCAGGTAA
COPS2_MBS2	WT	S	**TCGA**CAAAAGCGGCATTAAGCAGTTTCCAAAAGGTTTTGGAACTTGAAGGTGAAAAAG
		AS	**GGCC**CTTTTTCACCTTCAAGTTCCAAAACCTTTTGGAAACTGCTTAATGCCGCTTTTG
	MUT	S	**TCGA**CAAAAGCGGCATTAAGCAGTTTCCAAAAGGTTTTGGAAGTAGTAGGTGAAAAAG
		AS	**GGCC**CTTTTTCACCTACTACTTCCAAAACCTTTTGGAAACTGCTTAATGCCGCTTTTG
KPNA2	WT	S	**TCGA**TGGTTTGTTACTGTAGCACTTTTTACACTGAAACTATACTTGAACAGTTCCAAC
		AS	**GGCC**GTTGGAACTGTTCAAGTATAGTTTCAGTGTAAAAAGTGCTACAGTAACAAACCA
	MUT	S	**TCGA**TGGTTTGTTACTGTAGCACTTTTTACACTGAAACTATAGTAGTACAGTTCCAAC
		AS	**GGCC**GTTGGAACTGTACTACTATAGTTTCAGTGTAAAAAGTGCTACAGTAACAAACCA
MRPL15	WT	S	**TCGA**GGTACCTGGCGGATCCTGCCAAATTTCCTGAAGCACGACTTGAACTCGCCAGGA
		AS	**GGCC**TCCTGGCGAGTTCAAGTCGTGCTTCAGGAAATTTGGCAGGATCCGCCAGGTACC
	MUT	S	**TCGA**GGTACCTGGCGGATCCTGCCAAATTTCCTGAAGCACGAGTAGTACTCGCCAGGA
		AS	**GGCC**TCCTGGCGAGTACTACTCGTGCTTCAGGAAATTTGGCAGGATCCGCCAGGTACC
NOL12_MBS1	WT	S	**TCGA**TAATCCCAGCTACTTGGGAGGCTGAGGTGGAAGAATCACTTGAACCCTGGAGGC
		AS	**GGCC**GCCTCCAGGGTTCAAGTGATTCTTCCACCTCAGCCTCCCAAGTAGCTGGGATTA
	MUT	S	**TCGA**TAATCCCAGCTACTTGGGAGGCTGAGGTGGAAGAATCAGTAGTACCCTGGAGGC
		AS	**GGCC**GCCTCCAGGGTACTACTGATTCTTCCACCTCAGCCTCCCAAGTAGCTGGGATTA
NOL12_MBS2	WT	S	**TCGA**GAGCAGAGGAAGCTTCGGGAGGAGCGCCACCAGGAATACTTGAAGATGCTGGCA
		AS	**GGCC**TGCCAGCATCTTCAAGTATTCCTGGTGGCGCTCCTCCCGAAGCTTCCTCTGCTC
	MUT	S	**TCGA**GAGCAGAGGAAGCTTCGGGAGGAGCGCCACCAGGAATAGTAGTAGATGCTGGCA
		AS	**GGCC**TGCCAGCATCTACTACTATTCCTGGTGGCGCTCCTCCCGAAGCTTCCTCTGCTC

Green letters indicate miR-26b-5p binding sites and red letters indicate the mismatches introduced in the mutants.

© 2020 by the authors. Licensee MDPI, Basel, Switzerland. This article is an open access article distributed under the terms and conditions of the Creative Commons Attribution (CC BY) license (http://creativecommons.org/licenses/by/4.0/).
